# The Preparation of Primary Hematopoietic Cell Cultures From Murine Bone Marrow for Electroporation

**DOI:** 10.3791/1026

**Published:** 2009-01-06

**Authors:** Kelly Kroeger, Michelle Collins, Luis Ugozzoli

**Affiliations:** Gene Expression Division, Bio-Rad Laboratories, Inc

## Abstract

It is becoming increasingly apparent that electroporation is the most effective way to introduce plasmid DNA or siRNA into primary cells. The Gene Pulser MXcell electroporation system and Gene Pulser electroporation buffer were specifically developed to transfect nucleic acids into mammalian cells and difficult-to-transfect cells, such as primary and stem cells.This video demonstrates how to establish primary hematopoietic cell cultures from murine bone marrow, and then prepare them for electroporation in the MXcell system. We begin by isolating femur and tibia. Bone marrow from both femur and tibia are then harvested and cultures are established. Cultured bone marrow cells are then transfected and analyzed.

**Figure:**
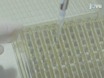


## Protocol

### Harvesting Bone Marrow From Femurs and Tibiae

The first step of the procedure is to harvest bone marrow from the femurs and tibiae of a 6- to 12-week old mouse (we are using BALB/c mice but you can do this procedure with any mouse strain). To begin, euthanize the mouse by CO_2_ inhalation (won’t be shown). Harvest femur and tibiae from the hind legs of each. Then, with a razor blade, cut each end of the femurs to remove the hip and knee joints and to expose the marrow.
In a similar manner, cut each end of the tibiae to remove the knee and ankle adjacent regions and to expose the marrow.
Take a 3 mL syringe with a 26-gauge needle and fill it with RPMI supplemented with 10% FBS, penicillin, streptomycin, and beta-mercaptoethanol (BME = 100 uM). Using tweezers, hold the bone over a Petri dish containing RPMI media. Insert the syringe needle into one end of the bone and depress the plunger to flush out the bone marrow. The syringe needle can be moved up and down inside the bone to flush out residual marrow.
Repeat the procedure for all the bones, and then proceed with establishing the cultures.

### Establishing Cultures

To establish the bone marrow cultures, pipette the flushed bone marrow up and down several times to break up the tissue into a cell suspension.
Place a 70 micron filter on top of a 50 mL conical tube, and add the cell suspension to the filter.
Rinse the Petri dish one time with RPMI, then add the rinse to the 70 micron filter.
Using the rubber end of a 1 mL syringe plunger, grind the bone marrow pieces remaining on top of the 70 micron filter.
Wash the filter one time with RPMI.
After washing the filter, centrifuge the cell suspension at 1200 rpm for 5 minutes.
Wash the cell pellet by resuspending in PBS.
Count the cells using a hemocytometer. We typically obtain ~90 million cells from one mouse’s bones (two femurs plus two tibiae). After counting the cells, centrifuge them again at 1200 rpm for 5 minutes.
Resuspend the cells in a small amount of RPMI and aliquot them into tissue culture flasks containing enough RPMI so that the final concentration is 1 million cells per mL.
Add cytokines to promote the development of your cell type of interest. In this case, the cytokine interleukin-3 (IL-3 = 10 ng/mL) is added to promote the development of basophils and mast cells. To acquire basophils, incubate for 10 days. For mast cells, incubate for 5 weeks. When generating mast cells, the media and IL-3 should be changed once a week.
Here is a look at how mast cells should appear just after plating. This image demonstrates the differentiation of mast marrow cells into basophils and mast cells 10 days after the addition of interleukin-3.
Once the desired cell type is obtained, proceed with the electroporation step.

 

### Electroporating Cells

To begin the electroporation step, determine the number of cells in the culture flask.
Cells are typically electroporated at a density of 10 million per mL, so transfer the required cell number to conical tubes and centrifuge the tubes at 1200 rpm for 5 minutes.
After centrifuging, aspirate the media, wash the cells with 1X PBS, and centrifuge again at 1200 rpm for 5 minutes.
Aspirate the PBS and add Bio-Rad Gene Pulser electroporation buffer to make a cell suspension of 10 million cells per mL.
After resuspending the cells, add the desired plasmid DNA at a final concentration of 10 to 20 micrograms per mL.
Aliquot 150 microliters of the cell suspension into wells of your choice on a 96-well electroporation plate.
Put the electroporation plate in the MXcell plate chamber and close the lid.
Prior to transfection of mast cells, an electroporation protocol must be programmed into the MXcell unless using a preset or stored protocol. Through optimization experiments we have discovered that the highest transfection efficiencies of mast cells occur using square wave pulse protocols. We will vary the electroporation conditions on the plate to deliver 300V/20ms, 350/15ms, 350V/20ms, and 350V/10ms square wave pulses at 2000uF and 1000 ohms. Once the protocol has been set and loaded onto the device, press “Pulse” to electroporate the cells.
After electroporation is complete, transfer the cells to a tissue culture plate. We typically transfer each 150 microliter electroporation sample to a well in a 48-well tissue culture plate containing 300 microliters RPMI (supplemented with 10% FCS, penicillin, streptomycin, and beta-mercaptoethanol) with 10 nanograms per mL interleukin-3. Cells are incubated overnight at 37°C, then assayed 24 hours later for expression and suppression.

### Transfection Results

The fluorescent microscopy image of the cells after successful electroporation using 20 micrograms per mL GFP plasmid is shown in the video. Using the MXcell electroporation system transfection efficiencies of about 30% can be obtained. The system allows you to vary conditions to maximize your transfection efficiency, while maintaining cell viability.

## Discussion

As more genomic information emerges and new tools such as siRNA are developed, the use of physiologically relevant cells has become ever more important to further our understanding of disease pathways, protein-protein interactions, and signal transduction. Primary cells are obtained directly from tissues or fluids and cultivated in vitro. These cells can be manipulated in a number of ways, including through the introduction of exogenous genetic material. It is becoming increasingly apparent that the most effective way to introduce plasmid DNA or siRNA into primary cells is electroporation.

Electroporation exposes cells to electric pulses in order to transiently increase the permeability of the cell membrane, thereby allowing exogenous nucleic acids to enter the cell. This method of transfection can be optimized to accommodate for differences between different cell types/lines and, thus, can be used to transfect mast cells as well as any other bone marrow-derived cell. Furthermore, unlike viral-mediated transfection, electroporation does not impose limits on the size of transfected DNA, nor require extensive preparation. In contrast to lipid-mediated transfection, electroporation can be less toxic and does not result in endosomal trapping of the transfected nucleic acid.

Bio-Rad has developed the MXcell electroporation system specifically for these cells. the MXcell allows you to vary conditions to maximize your transfection efficiency and cell viability. In order to optimize transfection efficiency and minimize cell death, a multitude of electroporation parameters including voltage, capacitance, resistance, and pulse length can be adjusted and evaluated.

